# Cooperative Intrinsic Basicity and Hydrogen Bonding Render SmI_2_ More Azaphilic than Oxophilic

**DOI:** 10.1021/acsomega.2c04680

**Published:** 2022-10-25

**Authors:** Gil Kolin, Renana Schwartz, Daniel Shuster, Dan Thomas Major, Shmaryahu Hoz

**Affiliations:** Department of Chemistry and Institute for Nanotechnology & Advanced Materials, Bar-Ilan University, Ramat Gan5290002, Israel

## Abstract

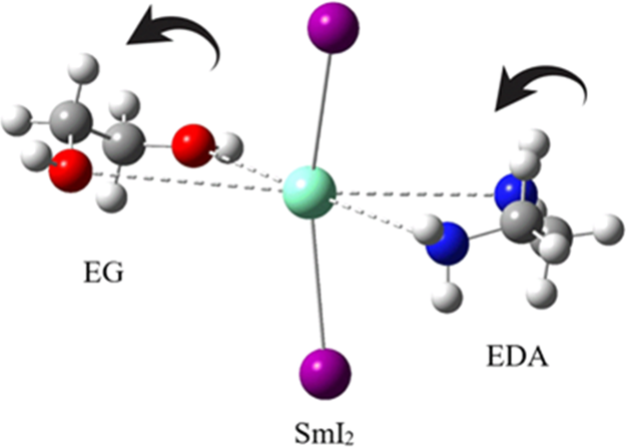

It has been recently shown that SmI_2_ is more azaphilic than oxophilic. Density functional theory calculations reveal that coordination of 1–3 molecules of ethylenediamine is more exothermic by up to 10 kcal/mol than coordination of the corresponding number of ethylene glycol molecules. Taking into account also hydrogen bonds between ligands and tetrahydrofuran doubles this preference. The intrinsic affinity parallels the order of basicity. The cooperativity with the hydrogen bonding makes SmI_2_ more azaphilic than oxophilic.

## Introduction

**Figure 1:**
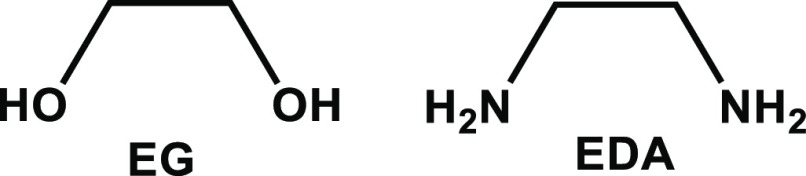
EG and EDA.

In SmI_2_ chemistry,^1^ the nature of its interaction with ligands is of crucial importance, as ligands strongly affect all major features of SmI_2_,^2^ including its reduction potential,^3^ chemoselectivity,^4^ stereoselectivity,^5^ reaction rate, and mechanism.^6^ It has been recently reported that SmI_2_, which was considered to be highly oxophilic, is in fact more azaphilic;^7^ namely, it has much higher affinity to aza ligands than to oxo ligands of similar structure. To elucidate the origin of this phenomenon, aza/oxo interactions of SmI_2_ were examined computationally with two simple bidentate ligands—ethylene glycol (EG) and ethylenediamine (EDA) (Figure 1).

## Computational Methods

The energetics of the addition of 1–3 EDA or EG ligand molecules to SmI_2_ were explored. Following the strategy devised by Maron,^8^ calculations were conducted at the meta-GGA functional M06 level^9^ using Gaussian 09.^10^ The Stuttgart–Dresden effective large-core basis set (4f-in-ECP, augmented by f polarization functions, α = 1.0) was used for samarium.^11^ Iodine atoms were represented by means of Stuttgart–Dresden effective core potentials in association with its basis set,^12^ augmented by d-polarization functions (α = 0.730).^13^ For the C, N, O, and H atoms, the 6-31G(d,p) basis set was used;^14^ for the oxygen atom of tetrahydrofuran (THF), the 6-31+G(d,p) basis set was used in cases where hydrogen bonding to this oxygen was considered. THF as a bulk solvent was treated as a continuum using the SMD implicit solvation model implemented in Gaussian 09.^15^ All complexes were constructed manually, and several starting structures were attempted and fully optimized. Following geometry optimization, the lowest energy structure was selected for further analysis. Basis set superposition errors were included using the counterpoise method in Gaussian 09. We note that more advanced approaches using molecular dynamics simulations have been performed.^8^

## Results and Discussion

The first mode of ligation studied was addition of ligands to SmI_2_, as shown in Eq 1 (L = EG; EDA)
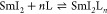
1

The results in Table 1 show that the complexation energies of EDA are significantly higher than those of EG.

**Table 1:** Equilibrium Energies (in kcal/mol) According to Eq 1

complex	L = EG	L = EDA	difference
SmI_2_(L)	–10.6	–13.6	3.0
SmI_2_(L)_2_	–21.6	–25.5	3.8
SmI_2_(L)_3_	–37.8	–39.8	2.1

**Figure 2:**
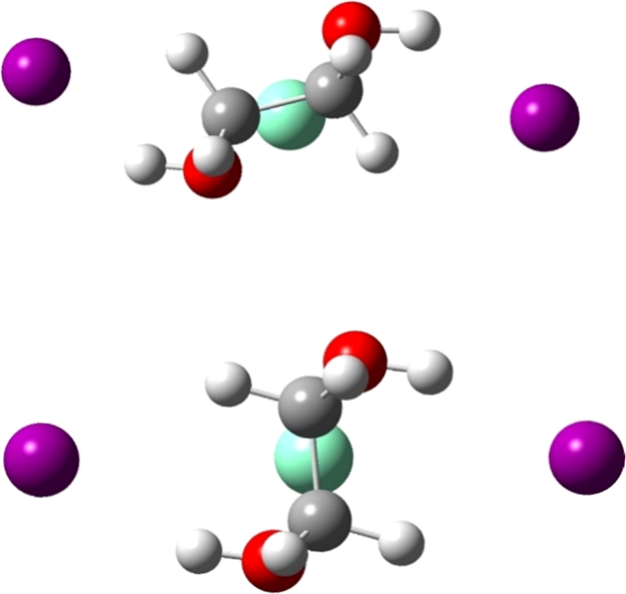
Two Orientations of SmI_2_ with One EG Ligand. Color Code: Sm—Cyan, I—Purple, C—Gray, O—Red, and H—White.

It should be noted that the potential surfaces of these complexes are very intricate. Even in the simple case of a single EG molecule coordinated to SmI_2_, there are at least two important geometries shown in Figure 2; the C–C bond is either almost parallel or orthogonal to the axis between the two iodides. For example, there is a difference of 1.1 kcal/mol between the two orientations (in favor of the orthogonal orientation), while for EDA there is no difference (0.0 kcal/mol).

In the case of SmI_2_(L)_3_, in addition to the two iodides, six oxygen or nitrogen atoms interact with the central samarium cation. The ligands can also interact among themselves and with the iodide ions through hydrogen bonds. The number of degrees of freedom is therefore very high. In each case, several (up to eight) initial geometries were used in order to increase the probability of attaining the lowest energy; in all cases, the two iodides were initially anti to each other. As it is not clear whether the global minimum was reached, our main focus is on energy trends.

We note that in the solid state as well as in solution, the sites on SmI_2_ are co-ligated by solvent and ligand molecules in a proportion that reflects their relative affinity and concentration.^1c^ In the solid state, five THF molecules can complex to SmI_2_,^1c^ while in solution, an equilibrium can exist for SmI_2_ with between 3 and 5 THF ligands.^1d^ Considering the aforementioned difficulty in finding the global minimum, a calculation where all of the SmI_2_ sites are occupied is computationally demanding. In addition, the two major energetic processes in replacing THF molecules by the ligand are the detachment of THF molecules and the consecutive association of the ligand to SmI_2_. All other interactions are energetically of the second order. Because EG and EDA are of similar size, the number of displaced THF molecules is highly likely to be similar for both ligands. Hence, the difference between EG and EDA in the energetics of the association to SmI_2_ will be nearly independent of the inclusion of the co-ligating THF molecules. Furthermore, because EDA is a better donor than EG, in the ligation of the second molecule of the additive, the detachment of THF molecules that precedes the ligation will be easier for EDA than for EG, accentuating further the differences between the two ligands.

The results in Table 1 show that EDA has a much higher affinity to SmI_2_ relative to EG. It is very likely that the intrinsic property that controls the ligand’s affinity to SmI_2_ is the same one that is responsible for the higher basicity of amine relative to the analogous oxygen compounds. This supposition is strengthened by considering the affinity of amines and alcohols to Sm^3+^. Due to its smaller size and larger charge, Sm^3+^ is much harder than Sm^2+^; hence, it resembles a proton much more than Sm^2+^, and much higher amine/alcohol preferential affinity is expected. While there is no direct comparison of EG and EDA affinity to Sm^3+^, it was shown by UV–vis spectroscopy that nitrogen ligands coordinate to Sm^3+^ much more efficiently than to SmI_2_ relative to oxygen ligands.^7^ This was also demonstrated by the effect of the two types of ligands on the reduction potential of SmI_2_. Namely, an increased affinity to Sm^3+^ over Sm^2+^ was reflected by a higher reduction potential according to Eq 2
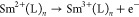
2

In general, amines increase the reduction potential of SmI_2_ more than alcohols. Due to solubility problems, the effect of EDA on the reduction potential could not be determined. Yet, while the reduction potential of SmI_2_ (2mM) increased from −1.13 (0 M) to −1.52 V (0.05 M) using EG as ligand,^3,7^ it increased further, to −1.6 V, with the sterically hindered EDA analogue *N*,*N*′-dimethylethylenediamine.^7^ Replacement of one OH group of EG by a MeNH unit increased the reduction potential much further to −1.85 V.^7^ Thus, it is clear that Sm^3+^ binds EDA much more than Sm^2+^ relative to EG, in accordance with the basicity model.

Another factor that may be involved in determining the affinities to SmI_2_ is hydrogen bonding. Both ligands carry groups capable of forming hydrogen bonds with THF before and after the binding to SmI_2_. In order to determine the effect of hydrogen bonding on the coordination energy of the two ligands, the equilibrium energies were computed according to Eq 3
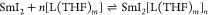
3

Calculations were performed for mono-, di-, and tri-ligation with one and two hydrogen bonds to THF for EG and one, two (on different nitrogen atoms), and in part three hydrogen bonds for EDA (*n* = *m* = 3 did not converge to a meaningful structure). The results are presented in Table 2.

**Table 2:** Effect of Hydrogen Bonding on Oxo/Azaphilicity of SmI_2_(in kcal/mol) According to Eq 3

*n*, *m*	EG	EDA
1, 1	–9.9	–21.2
1, 2	–11.1	–21.9
1, 3		–19.4
2, 1	–20.0	–41.4
2, 2	–23.4	–41.2
2, 3		–35.7
3, 1	–41.6	–55.9
3, 2	–46.5	–58.7

Table 3 compares the effect (Δ*E*) of the hydrogen bonding on the preference of SmI_2_ for EDA over EG according to Eq 4
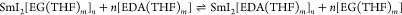
4

The data in Table 3 clearly shows that hydrogen bonding significantly enhances the azaphilicity over the oxophilicity. As the number of hydrogen bonds increases, the differences between the aza- and oxophilicity increase at all levels of ligation. In fact, hydrogen bonding contributes more than 50% of the overall difference between the azaphilicity and oxophilicity of SmI_2_.

**Table 3:** Preference of SmI_2_ for EDA Over EG (Δ*E*) as a Function of the Number of Hydrogen Bonds and Level of Coordination

	Δ*E*(kcal/mol)		
	*n*, numberof ligands		
*m*, numberof hydrogen bonds	1	2	3
0	–3.0	–3.8	–2.1
1	–11.3	–21.4	–14.3
2	–10.8	–17.8	–12.2

The enhanced hydrogen bonding of EDA over EG is somewhat surprising. According to the basicity model, as H_3_O^+^ is more acidic than NH_4_^+^ and as the hydrogen bonding efficiency is expected to parallel the acidity,^16^ EG is expected to form stronger hydrogen bonds than EDA. It seems likely that the primary cause for the hydrogen bonding supremacy of EDA stems from the different intrinsic bonding to the samarium cation. The amine binds more firmly to SmI_2_ and therefore acquires more onium character than the corresponding alcohol and becomes more acidic. This higher acidity causes in turn a more efficient hydrogen bonding. In fact, this is an auto-enhancing process^17^ by which the stronger binding enhances the hydrogen bonding, which in turn further enhances the binding to the samarium by releasing more electrons from the N–H bond to the nitrogen atom. Such cooperativity is widely recognized in many systems.^18^ In terms of classical physical organic chemistry, the energy gain here is similar to the initial stage of general base catalysis of nucleophilic attacks, where deprotonation of the nucleophile enhances its nucleophilicity.^19^

## Conclusions

In conclusion, the supremacy of EDA over EG in binding to SmI_2_ originates, most probably, from the same source that makes amines more basic than the corresponding alcohols. Calculations of the effect of hydrogen bonding to THF solvent molecules show that the differential gain in energy due to hydrogen bonding is equivalent to the intrinsic “basicity” effect. The higher energy gain associated with hydrogen bonding by EDA originates from its stronger binding to the samarium cation, which in turn increases its “onium” character to a greater extent than EG. Consequently, due to this cooperative effect, EDA forms stronger hydrogen bonds to THF than EG, which more than doubles the amount of energy gained in its coordination to SmI_2_. The current understanding may help in designing SmI_2_ reactions or SmI_2_-mediated reactions.

## Supporting Information Available

Cartesian coordinates and absolute energy of complexes (PDF)
