# Supplementary material for: Cooperative Intrinsic Basicity and Hydrogen Bonding Render SmI2 More Azaphilic than Oxophilic
Source: ACS Omega. 2022 Oct 25;7(44):40021–4. doi: 10.1021/acsomega.2c04680 (PMC9647864; doi:10.1021/acsomega.2c04680)
Supplement: Supplementary file 1 — ao2c04680_si_001.pdf [file ao2c04680_si_001.pdf]

Supporting Information for

Cooperative Intrinsic Basicity and Hydrogen Bonding Render SmI_2 More Azaphilic than Oxophilic

Gil Kolin, Renana Schwartz, Daniel Shuster, Dan Thomas Major* and Shmaryahu Hoz*

Department of Chemistry and Institute for Nanotechnology & Advanced Materials, Bar-Ilan University, Ramat Gan 5290002, Israel

shoz@mail.biu.ac.il

majort@mail.biu.ac.il

Energies (a.u.) and coordinates.

Table S1. Cartesian coordinates for SmI_2 complexes.

SmI_2

SCF ENERGY=-51.8861311

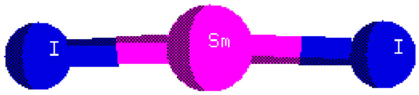

Sm	0	0	0	0
I	0	0	0	3.554253
I	0	0	0	-3.55425

EG

SCF ENERGY=-230.1304342

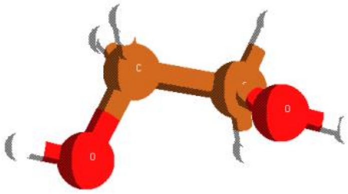

C	-1.6214	0.964386	0.026015
H	-1.32268	1.556351	-0.85654
H	-2.7242	0.934201	0.043013
C	-1.14479	1.684181	1.255997
H	-0.04385	1.622025	1.293828
H	-1.4102	2.750932	1.155037
O	-1.06311	-0.3294	0.001208
H	-1.44123	-0.7987	-0.75053
O	-1.74087	1.102332	2.392724
H	-1.36459	1.526299	3.171826

EDA

SCF ENERGY= -190.3992966

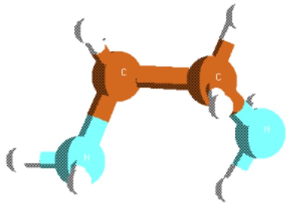

N	1.480056	-0.49724	-0.26054
N	-1.45035	-0.55678	0.045749
C	0.68269	0.60419	0.267506
C	-0.73824	0.677943	-0.27146
H	0.636751	0.505053	1.363894
H	1.202055	1.550542	0.066983
H	-0.69821	0.778249	-1.36634
H	-1.22935	1.590866	0.1125
H	-1.63815	-0.58459	1.045971
H	-2.36005	-0.56067	-0.40701
H	1.607676	-0.3497	-1.26042
H	0.905464	-1.3367	-0.19292

TSCF ENERGY

SCF ENERGY=-232.3011124

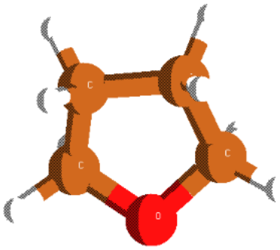

C	-1.11939	-0.45378	-0.12027
O	-8.9E-06	-1.16443	0.372895
C	1.119386	-0.4538	-0.12027
C	0.770859	1.027193	0.036737
C	-0.77084	1.027206	0.036737
H	-1.28612	-0.69991	-1.18526
H	-2.00422	-0.7681	0.44337
H	2.004212	-0.76814	0.44337
H	1.286111	-0.69993	-1.18526
H	1.159704	1.419838	0.982283
H	1.197105	1.63137	-0.77051
H	-1.15968	1.419858	0.982284
H	-1.19708	1.63139	-0.77051

SmI2_EG_1

SCF ENERGY=-282.0370007

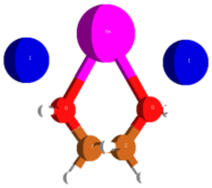

Sm	0.091602	-0.86435	0.030259
I	-3.35981	0.241252	0.174654
I	3.661636	-0.25647	-0.17332
C	0.428417	2.653972	0.658177
H	0.056678	3.604776	1.06299
H	1.493557	2.578115	0.911504
C	0.238934	2.615882	-0.83735
H	0.735985	3.476747	-1.30419
H	-0.82769	2.670153	-1.08994
O	-0.21298	1.546922	1.290003
H	-1.17767	1.655564	1.200073
O	0.718981	1.390728	-1.38937
H	1.689877	1.371649	-1.30277

SmI_2_EG_2

SCF ENERGY=-512.1888121

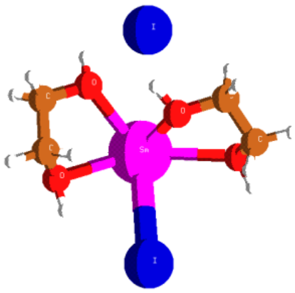

Sm	-0.59421	-0.04597	-1.11537
I	2.791906	0.048032	-2.05625

I	-3.88296	0.285985	0.405568
C	-0.40662	-2.7457	1.404723
H	-0.27393	-3.0232	2.458804
H	-1.05359	-3.49544	0.928302
C	0.926555	-2.71343	0.719936
H	1.428393	-3.6817	0.851021
H	1.563969	-1.93345	1.168248
C	-0.14651	2.38188	1.406511
H	0.266012	2.74833	2.356095
H	-1.23657	2.539415	1.417495
C	0.475177	3.137096	0.271304
H	1.560577	2.958908	0.258206
H	0.296046	4.212443	0.40214
O	-0.97987	-1.4463	1.287943
H	-1.94162	-1.47682	1.42517
O	0.70506	-2.4366	-0.65984
H	1.562904	-2.23456	-1.07319
O	-0.11526	2.668372	-0.94303
H	0.376543	3.049318	-1.6826
O	0.158421	1.006612	1.209531
H	-0.30288	0.444264	1.849559

SmI_2_EG_3

SCF ENERGY=-742.3494295

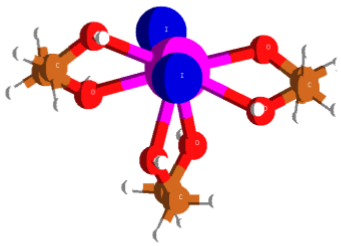

Sm	0.090861	-0.8906	0.027766
C	0.878668	0.050659	3.376405
H	1.180354	0.775366	4.144845
H	1.417715	-0.89108	3.561906
C	-0.59956	-0.18516	3.470082
H	-1.14193	0.74954	3.267717
H	-0.8464	-0.51915	4.487135
C	0.826734	-1.32954	-3.36951

H	1.216072	-0.29941	-3.33003
H	1.2443	-1.82192	-4.25835
C	-0.66945	-1.31036	-3.46963
H	-1.05228	-2.34199	-3.45054
H	-0.9643	-0.8517	-4.42369
C	-1.3706	2.279454	-0.52486
H	-1.95204	3.145811	-0.18149
H	-1.64638	2.05987	-1.56594
C	0.091006	2.604811	-0.44355
H	0.36821	2.791599	0.603888
H	0.291533	3.512585	-1.02854
O	-1.61433	1.146123	0.306813
H	-2.51531	0.820263	0.15432
O	0.818469	1.494766	-0.9639
H	1.769259	1.628417	-0.8261
O	1.166281	0.541666	2.069468
H	2.12672	0.5226	1.924494
O	-0.93845	-1.18077	2.510999
H	-1.8996	-1.29872	2.469394
O	1.17747	-2.03802	-2.18328
H	2.130878	-1.92693	-2.0233
O	-1.18901	-0.56768	-2.37066
H	-2.0832	-0.89238	-2.16913
I	-3.40333	-2.07008	-0.02583
I	3.763532	-0.53222	-0.24609

SmI_2_EDA_1

SCF ENERGY=-242.3140416

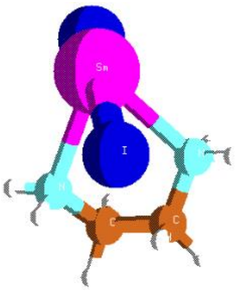

Sm	0.031269	-0.92049	0.009139
I	-3.34325	-0.34088	-0.99414
I	3.405044	-0.33063	1.009289
C	0.226766	2.566727	0.728449

H	-0.07518	3.524579	1.178159
H	1.321717	2.504104	0.809473
C	-0.16857	2.561744	-0.73269
H	0.132132	3.517068	-1.18857
H	-1.26346	2.497205	-0.81335
H	-1.35238	1.558044	1.536762
H	1.411718	1.550226	-1.53547
N	-0.34847	1.410987	1.431808
N	0.408133	1.402284	-1.42865
H	0.025337	1.376888	2.378144
H	0.033582	1.361138	-2.37441

SmI_2_EDA_2

SCF ENERGY-432.7392002

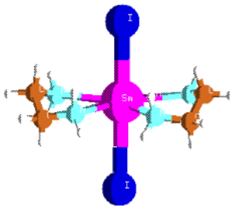

Sm	0.054213	-0.00017	-0.7019
I	3.510495	-0.35291	0.055997
I	-3.44888	0.362161	-0.23505
C	0.089794	-3.51526	0.102665
C	-0.68849	-2.95816	1.276306
H	-1.76013	-2.955	1.030045
H	-0.5618	-3.62796	2.140872
H	-0.10477	-4.59651	0.026628
H	1.167195	-3.39622	0.293026
C	0.622661	3.006374	1.173247
C	-0.31748	3.504969	0.095571
H	0.579225	3.69034	2.034834
H	1.655138	3.034925	0.794541
H	-1.35586	3.324179	0.4098
H	-0.19866	4.594149	-0.01207
N	-0.24086	-2.79439	-1.13402
H	0.319327	-3.16819	-1.89704
H	-1.20797	-3.00365	-1.38085
N	-0.28435	-1.57875	1.564225
H	-0.93375	-1.16034	2.226877
H	0.624614	-1.58577	2.026963
N	-0.09422	2.786567	-1.16582

H	-0.81999	3.045567	-1.8308
H	0.779844	3.113908	-1.57602
N	0.311709	1.618781	1.531096
H	1.033064	1.255504	2.15109
H	-0.55639	1.597568	2.065168

SmI_2_EDA_3

SCF ENERGY= -623.169694

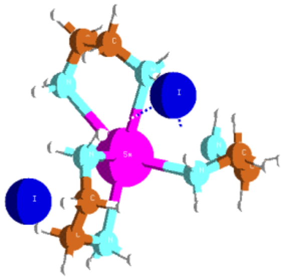

Sm	-0.77798	-0.65404	-0.09406
I	4.311433	0.840315	0.110796
I	-4.16482	0.680728	0.050802
N	0.529719	1.693814	0.616661
H	1.489674	1.674851	0.26569
N	-1.19705	0.261488	2.580886
C	0.54847	1.916375	2.068412
C	-0.82309	1.680805	2.667155
H	1.267756	1.21127	2.511228
H	0.89421	2.926435	2.335788
H	-1.5692	2.257726	2.100843
H	-0.83976	2.061106	3.699767
H	0.080139	2.49144	0.167764
H	-0.67067	-0.26524	3.276481
H	-2.17882	0.154271	2.8294
N	1.491715	-1.53703	1.29213
H	2.225227	-0.8317	1.187483
N	-0.09658	-3.4569	-0.2996
C	1.996727	-2.83374	0.827526
C	0.874147	-3.84551	0.733938
H	2.442837	-2.69007	-0.16777
H	2.793803	-3.23619	1.471145
H	0.344648	-3.88653	1.696718
H	1.300517	-4.84542	0.561233
H	1.306495	-1.5942	2.292859
H	0.323913	-3.59911	-1.21746
H	-0.89715	-4.08479	-0.26937
N	-1.22452	0.805955	-2.51125

H	-2.14065	0.6404	-2.92297
N	1.343337	-0.48384	-1.92442
C	-0.17055	0.616065	-3.51493
C	1.196581	0.635543	-2.86376
H	-0.32826	-0.36175	-3.99281
H	-0.20111	1.369727	-4.31661
H	1.319061	1.572223	-2.29941
H	1.971838	0.638845	-3.6452
H	-1.2392	1.781767	-2.21513
H	1.468686	-1.34476	-2.45645
H	2.210915	-0.35921	-1.39589

EG_1_TSCF ENERGY_1

SCF ENERGY=-462.4445061

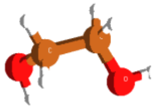

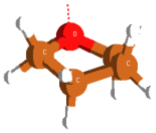

C	-0.06985	1.227733	2.159171
H	0.080662	1.470118	3.22549
H	0.789341	0.621975	1.827025
C	-0.10708	2.50258	1.355671
H	-0.99178	3.087482	1.67201
H	0.779684	3.101688	1.604605
O	-0.09786	2.277303	-0.02624
H	-0.95451	1.879886	-0.27058
O	-1.29127	0.54659	1.949737
H	-1.22472	-0.32811	2.347458
O	-2.67086	1.7332	-0.92406
C	-3.19065	3.072684	-0.87824
H	-2.43606	3.710233	-0.39461
H	-3.33456	3.43721	-1.90307
C	-3.56988	0.853487	-0.2404
H	-4.26541	0.400681	-0.96595
H	-2.97079	0.067294	0.22892
C	-4.48723	3.007264	-0.07999
H	-4.64821	3.905672	0.52369

H	-5.34688	2.893932	-0.75297
C	-4.29981	1.738483	0.745221
H	-3.65053	1.924838	1.610861
H	-5.24029	1.30397	1.098286

EG_1_TSCF ENERGY_2

SCF ENERGY=-694.7606279

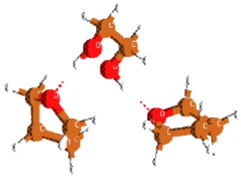

O	0.32797	-0.06194	0.620116
O	0.412887	2.038194	-1.42055
C	0.122073	2.203249	-0.06064
H	-0.52789	3.084605	0.036011
H	1.032137	2.40863	0.533655
C	-0.57738	1.013053	0.549858
H	-1.4539	0.759932	-0.07529
H	-0.95762	1.291799	1.550811
H	1.091445	1.343234	-1.51413
H	-0.19329	-0.88252	0.689303
O	2.223851	0.00333	-2.01178
O	-1.40712	-2.2089	0.543715
C	-2.46647	-2.25199	1.512769
H	-2.15626	-2.91138	2.330586
H	-2.62477	-1.24042	1.921219
C	-1.95919	-2.27128	-0.7818
H	-1.49466	-1.48445	-1.39142
H	-1.70579	-3.24725	-1.22231
C	3.134623	-0.46067	-1.00641
H	4.097817	0.035936	-1.16826
H	2.744925	-0.18674	-0.01435
C	1.367823	-1.07491	-2.41698
H	0.323552	-0.74796	-2.312
H	1.559213	-1.28448	-3.47947
C	3.174868	-1.96543	-1.17658
H	3.517369	-2.48368	-0.27555
H	3.83918	-2.23914	-2.00733
C	1.721449	-2.26033	-1.53373
H	1.10413	-2.25677	-0.62614
H	1.577857	-3.21798	-2.04441
C	-3.68655	-2.73343	0.754004

H	-3.67958	-3.82854	0.674035
H	-4.6241	-2.42908	1.228705
C	-3.45762	-2.10087	-0.61359
H	-3.71997	-1.03467	-0.58656
H	-4.02619	-2.57318	-1.42014

EDA_1_TSCF ENERGY_1

SCF ENERGY=-422.6960707

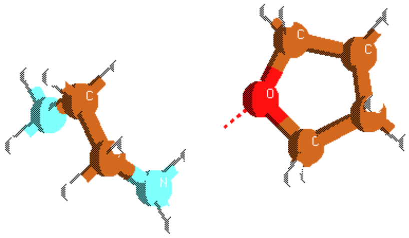

N	0.829061	-0.26697	2.308951
H	0.666652	-0.88636	3.098133
N	0.000305	1.445886	0.045874
C	0.011588	0.931799	2.457673
C	0.298323	1.959231	1.378171
H	-1.04845	0.637253	2.39163
H	0.129309	1.435003	3.435757
H	1.36571	2.230258	1.420205
H	-0.264	2.875473	1.638614
H	1.810776	-0.00353	2.362265
H	-0.99643	1.230637	0.015383
H	0.13934	2.185851	-0.63801
C	-3.15984	2.554509	-1.08996
O	-3.05681	2.039466	0.24153
C	-4.35365	1.660437	0.711073
C	-5.24466	1.624576	-0.51652
C	-4.64263	2.749804	-1.35042
H	-2.57547	3.480993	-1.15596
H	-2.7265	1.824502	-1.79357
H	-4.27076	0.69657	1.227594
H	-4.71003	2.409195	1.436708
H	-5.13787	0.6643	-1.03858
H	-6.30249	1.769381	-0.27648
H	-4.89325	2.697084	-2.4143
H	-4.97408	3.723863	-0.96661

EDA_1_TSCF ENERGY_2

SCF ENERGY=-655.0057907

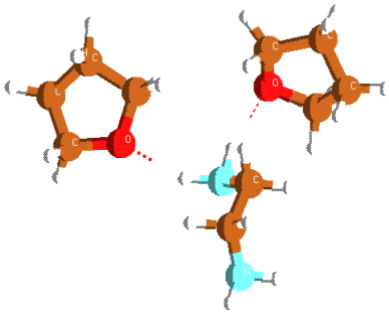

N	-0.0365	-1.2629	2.119751
H	-0.5116	-1.8629	2.788351
N	0.136343	0.947704	0.161693
C	-0.2932	0.133282	2.452253
C	0.49005	1.085486	1.567183
H	-1.36721	0.332735	2.310392
H	-0.06643	0.391484	3.503989
H	1.567376	0.872982	1.68487
H	0.337499	2.105896	1.974691
H	0.956983	-1.45202	2.234671
H	-0.86569	1.115729	0.067617
H	0.605145	1.670491	-0.38565
C	3.133373	2.456246	-0.88167
O	1.889977	3.106483	-1.17761
C	1.741355	4.257073	-0.33962
C	3.110147	4.512411	0.261234
C	3.638215	3.089585	0.401912
H	2.953816	1.375595	-0.80371
H	3.833005	2.627263	-1.71434
H	1.356398	5.084978	-0.94692
H	1.002212	4.038123	0.450561
H	3.735638	5.084051	-0.43736
H	3.059633	5.061386	1.206418
H	4.725784	3.026907	0.504369
H	3.180253	2.60175	1.274411
C	-2.12843	3.640913	0.235987
O	-2.66644	2.325465	0.39811

C	-4.00279	2.287084	-0.11551
C	-4.17721	3.563312	-0.9177
C	-3.30259	4.53074	-0.12932
H	-1.6322	3.937441	1.169071
H	-1.37487	3.626888	-0.57004
H	-4.12482	1.37273	-0.7079
H	-4.71238	2.248611	0.726336
H	-3.78132	3.434869	-1.93394
H	-5.22374	3.874098	-0.99342
H	-2.99983	5.417181	-0.69502
H	-3.82633	4.863179	0.777002

EDA_1_TSCF ENERGY_3

SCF ENERGY=-887.3174925

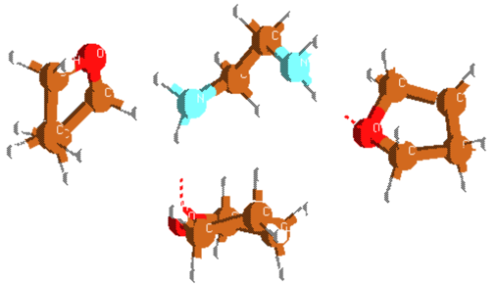

N	0.781439	-0.98279	1.236815
H	1.049431	-1.92519	1.507099
N	-1.18101	1.144489	0.456634
C	-0.30975	-0.50907	2.081572
C	-0.62899	0.948659	1.794576
H	-1.20434	-1.12035	1.884681
H	-0.10316	-0.59998	3.165803
H	0.311497	1.519586	1.874055
H	-1.28938	1.325698	2.597954
H	1.589966	-0.38525	1.406592
H	-2.18432	0.971548	0.487391
H	-1.073	2.117956	0.174373
C	0.671664	2.926927	-1.71882
O	-0.02101	3.761804	-0.78789
C	0.846817	4.064366	0.31175
C	2.150552	3.307456	0.071819
C	1.731618	2.211855	-0.90604
H	-0.06328	2.254989	-2.17878

H	1.123767	3.546366	-2.51153
H	0.994572	5.151027	0.371069
H	0.347379	3.744111	1.240067
H	2.893225	3.966715	-0.39609
H	2.574309	2.91346	1.002337
H	2.559392	1.844068	-1.5215
H	1.279341	1.357561	-0.38238
C	-3.59675	3.427979	1.363625
O	-4.13706	2.124798	1.124843
C	-4.60962	2.044019	-0.22404
C	-3.98904	3.22205	-0.95019
C	-3.96977	4.270919	0.155619
H	-3.99993	3.811204	2.30898
H	-2.50055	3.348029	1.464596
H	-4.323	1.068336	-0.63722
H	-5.70977	2.108058	-0.23045
H	-2.96394	2.980406	-1.26578
H	-4.55939	3.522286	-1.83463
H	-3.26273	5.087398	-0.02154
H	-4.97087	4.704307	0.283177
C	4.138854	0.418613	1.800214
O	2.974936	0.947396	2.424249
C	2.919756	0.3065	3.693571
C	4.3638	0.256188	4.192392
C	5.196729	0.36518	2.899095
H	3.92256	-0.59416	1.412979
H	4.393755	1.06322	0.951943
H	2.236284	0.877633	4.330428
H	2.509849	-0.71289	3.570343
H	4.574039	1.095059	4.863997
H	4.562136	-0.66685	4.746369
H	5.797004	1.281204	2.899533
H	5.880346	-0.47823	2.760429

SmI_2_EG_1_TSCF ENERGY_1

SCF ENERGY=-514.3513826

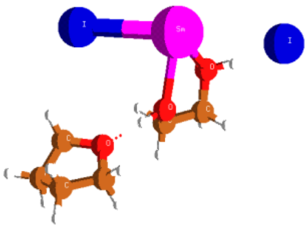

C	-0.44301	-0.52549	2.418569
H	-0.59688	-0.18932	3.452961
H	-0.88993	-1.52471	2.307711
C	1.025726	-0.594	2.121924
H	1.473087	0.412258	2.205396
H	1.51372	-1.24537	2.861062
O	1.193621	-1.10705	0.807695
H	2.142109	-1.34077	0.691994
O	-1.05334	0.384413	1.502669
H	-1.95526	0.066913	1.324145
O	3.824327	-1.69189	0.640963
C	4.406971	-3.00596	0.649386
H	4.133768	-3.51396	1.586514
H	3.990651	-3.57437	-0.18951
C	4.837378	-0.70249	0.908506
H	5.03189	-0.1412	-0.01702
H	4.454146	-0.00495	1.662699
C	5.901942	-2.77788	0.561724
H	6.477444	-3.61512	0.967357
H	6.20468	-2.62353	-0.48241
C	6.056937	-1.48529	1.354782
H	6.008199	-1.69237	2.431854
H	6.989793	-0.95186	1.150264
Sm	-0.03905	0.391734	-1.05159
I	-3.15853	-1.41946	-0.68502
I	2.634771	2.610392	-0.39019

SmI_2_EG_1_TSCF ENERGY_2

SCF ENERGY=-746.6683174

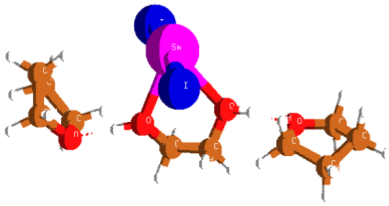

C	-0.4514	-0.43534	2.369509
H	-0.58048	-0.13857	3.419753
H	-1.08388	-1.31998	2.181803
C	0.987832	-0.78669	2.129844
H	1.621128	0.095655	2.318667
H	1.286539	-1.5821	2.828299
O	1.132344	-1.22308	0.784688
H	2.076461	-1.46371	0.653603
O	-0.81507	0.630347	1.499922
H	-1.64418	1.032356	1.845749
O	3.780511	-1.71631	0.670339
C	4.512986	-2.95295	0.703327
H	4.24036	-3.50445	1.61552
H	4.22127	-3.55263	-0.16606
C	4.659503	-0.61412	0.963931
H	4.845254	-0.05326	0.036051
H	4.155315	0.053324	1.673041
C	5.974203	-2.55173	0.713227
H	6.614305	-3.31621	1.163372
H	6.326687	-2.3653	-0.30966
C	5.927891	-1.24749	1.50072
H	5.829257	-1.45436	2.574516
H	6.805728	-0.61228	1.351793
O	-3.16028	1.578112	2.45703
C	-4.12213	0.511318	2.2951
H	-3.57934	-0.38276	1.950822
H	-4.57374	0.292888	3.269542
C	-3.5499	2.685949	1.631441
H	-2.6384	3.211414	1.322251
H	-4.16898	3.381267	2.218544
C	-4.34121	2.060849	0.504366
H	-4.97337	2.779421	-0.02556
H	-3.66018	1.59272	-0.22275
C	-5.12701	0.991783	1.256376
H	-6.00187	1.441876	1.742253
H	-5.47242	0.174381	0.614708
Sm	-0.24291	0.121169	-1.08462
I	-3.13409	-1.99947	-0.87975

I	2.420404	2.436214	-0.78478

SmI_2_EDA_1_TSCF ENERGY_1

SCF ENERGY=-474.6244309

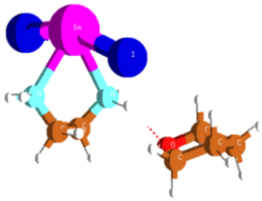

Sm	0.086204	0.226904	-1.9111
I	-3.35564	1.085175	-1.49362
I	3.542791	-0.32291	-1.25994
N	-0.36603	-1.16736	0.41616
H	-1.14731	-1.81739	0.354731
N	0.256874	1.732624	0.397072
C	-0.58644	-0.26119	1.554654
C	0.429425	0.861967	1.569135
H	-1.59576	0.164868	1.454894
H	-0.55365	-0.78897	2.519588
H	1.445979	0.441506	1.548532
H	0.323422	1.417675	2.513663
H	0.45272	-1.74299	0.612712
H	-0.62066	2.240648	0.539573
H	0.989073	2.440565	0.400071
C	-1.65277	4.623676	1.193645
O	-1.91103	3.338317	1.766792
C	-3.32034	3.188368	1.994565
C	-3.983	4.368592	1.3077
C	-2.90727	5.440822	1.432453
H	-0.75273	5.039292	1.663063
H	-1.45921	4.512725	0.111407
H	-3.64106	2.218323	1.592324
H	-3.50343	3.198248	3.080027
H	-4.16511	4.13428	0.249656
H	-4.93677	4.642231	1.769003
H	-3.01755	6.26329	0.719151
H	-2.90092	5.861451	2.447049

SmI$_2$_EDA_1_TSCF ENERGY_2

SCF ENERGY=-706.9320416

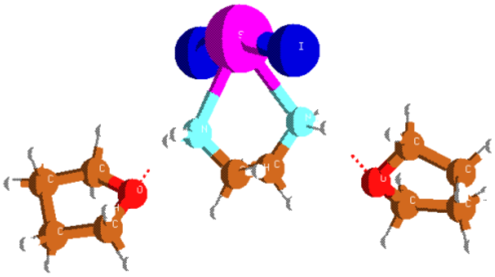

Sm	-0.25007	-0.15328	-1.84386
I	-3.64257	-0.50964	-0.99396
I	3.2873	0.051691	-1.92655
N	0.087156	-1.09839	0.733484
H	-0.46534	-1.93044	0.931582
N	-0.07149	1.789927	0.089413
C	-0.25391	-0.05016	1.706836
C	0.442573	1.2477	1.356926
H	-1.34264	0.104086	1.689339
H	0.01941	-0.33584	2.735186
H	1.521378	1.062028	1.251394
H	0.315698	1.956502	2.191099
H	1.06229	-1.35633	0.908698
H	-1.006	2.163278	0.286635
H	0.496563	2.589766	-0.18299
C	-1.78132	4.158443	2.097806
O	-2.39355	2.96367	1.595212
C	-3.67745	3.28044	1.040899
C	-3.67027	4.781072	0.831632
C	-2.84479	5.241095	2.027414
H	-1.4137	3.968804	3.113883
H	-0.91492	4.408666	1.462469
H	-3.81051	2.696319	0.121944
H	-4.46366	2.980328	1.751393
H	-3.15829	5.034194	-0.10685
H	-4.67716	5.20806	0.799609
H	-2.41792	6.242202	1.914093
H	-3.46083	5.231939	2.936213
C	3.541303	-2.37843	1.479824
O	2.694532	-1.58035	2.300129
C	2.158594	-2.47881	3.267024
C	3.270162	-3.48866	3.582557
C	4.294764	-3.267	2.45541
H	2.925121	-2.9835	0.786347

H	4.170561	-1.70874	0.88492
H	1.832402	-1.88886	4.129657
H	1.274884	-2.98926	2.842931
H	3.713865	-3.30332	4.565755
H	2.877665	-4.51082	3.58754
H	5.177529	-2.73828	2.832141
H	4.634505	-4.19886	1.992977

SmI_2_EDA_1_TSCF ENERGY_3

SCF ENERGY=-939.2435754

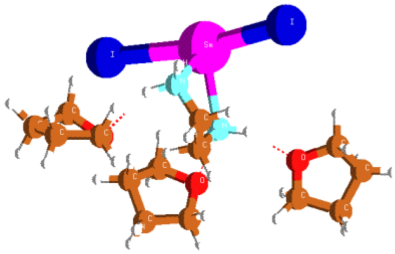

Sm	-0.11845	0.025369	-1.85745
I	-3.60186	-0.57673	-1.21852
I	3.385288	-0.48295	-2.20724
N	0.088136	-1.17184	0.611533
H	-0.45613	-2.03046	0.673757
N	0.098958	1.764932	0.263993
C	-0.37538	-0.23044	1.640429
C	0.3743	1.081083	1.53585
H	-1.45264	-0.0586	1.493424
H	-0.24599	-0.63041	2.659222
H	1.452936	0.874675	1.598812
H	0.113712	1.706581	2.406459
H	1.05071	-1.42426	0.850078
H	-0.81879	2.211075	0.360312
H	0.766981	2.532251	0.141095
C	3.33212	3.428341	-0.39817
O	2.168824	4.019586	0.182926
C	2.368809	4.123668	1.600216
C	3.627669	3.318675	1.934085
C	3.863228	2.496392	0.668591

H	3.028203	2.925847	-1.32521
H	4.068506	4.209568	-0.65022
H	2.463704	5.181726	1.879656
H	1.468125	3.726727	2.091701
H	4.475803	3.991266	2.110837
H	3.499301	2.700912	2.828881
H	4.911128	2.220453	0.513702
H	3.270581	1.569839	0.680937
C	-1.69335	4.277947	1.92627
O	-2.30165	3.077585	1.437835
C	-3.64673	3.351369	1.020397
C	-3.76345	4.863326	0.974167
C	-2.82617	5.272118	2.104655
H	-1.1569	4.04754	2.855713
H	-0.96162	4.642691	1.1841
H	-3.81799	2.858822	0.055415
H	-4.34279	2.917031	1.754392
H	-3.39169	5.24787	0.014993
H	-4.79266	5.209998	1.106872
H	-2.48729	6.310868	2.046435
H	-3.31556	5.122304	3.076092
C	3.580223	-2.24114	1.573645
O	2.61877	-1.52924	2.346414
C	2.079809	-2.48711	3.254235
C	3.223916	-3.44948	3.604137
C	4.320956	-3.09949	2.584414
H	3.06521	-2.86798	0.820501
H	4.202519	-1.51101	1.045754
H	1.674809	-1.94266	4.113556
H	1.249774	-3.02876	2.766338
H	3.572668	-3.30766	4.631758
H	2.897018	-4.49032	3.510018
H	5.116466	-2.51149	3.056233
H	4.78148	-3.98068	2.127274

SmI_2_EG_1_TSCF ENERGY_1

SCF ENERGY=-514.3513826

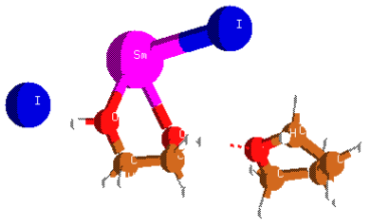

C	0.85808	1.10947	2.4777
H	0.81256	1.07891	3.57481
H	1.4546	1.98428	2.1788
C	-0.52797	1.22847	1.91683
H	-1.12201	0.33896	2.19169
H	-1.01849	2.11196	2.34994
O	-0.4308	1.34637	0.50429
H	-1.30965	1.62571	0.16188
O	1.45672	-0.08357	1.97017
H	2.41096	0.07934	1.8752
O	-2.89949	2.10614	-0.28235
C	-3.28325	3.41426	-0.73826
H	-3.07719	4.1465	0.0569
H	-2.6741	3.66839	-1.61262
C	-4.07145	1.34105	0.06105
H	-4.20973	0.55398	-0.69452
H	-3.90395	0.86247	1.03323
C	-4.76735	3.31511	-1.02504
H	-5.26922	4.28578	-0.97364
H	-4.93641	2.8906	-2.02348
C	-5.21765	2.33372	0.05099
H	-5.29451	2.84244	1.02086
H	-6.17824	1.85587	-0.16239
Sm	0.8281	-0.74364	-0.62256
I	4.08968	0.78709	-0.21644
I	-2.20253	-2.40263	0.1339

SmI_2_EG_2_TSCF ENERGY_1

SCF ENERGY=-744.5063326

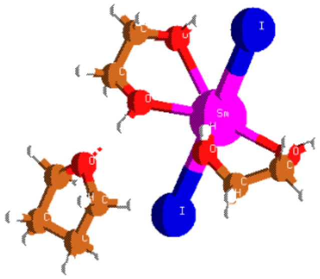

Sm	0.926257554	1.018028161	0.988056
I	3.691898831	-0.21974074	2.705908
I	-1.920335859	1.358850819	-1.15622
C	-1.177630172	-1.84523144	1.785925
H	-1.540927278	-2.52128229	2.572414
H	-1.945435514	-1.78774504	0.998401
C	0.107545354	-2.38383723	1.221587
H	-0.050234641	-3.40059623	0.839219
H	0.876866592	-2.43057401	2.003216
O	-0.913802748	-0.54652283	2.316931
H	-1.747251817	-0.16483941	2.62141
O	0.635999138	-1.53130012	0.209285
H	0.072485819	-1.58081967	-0.57742
O	2.24778173	0.405421746	-1.19602
O	1.240218609	2.981833101	-0.98473
C	1.924920896	2.541678288	-2.15874
H	2.426184296	3.390232685	-2.64351
H	1.206990783	2.109293385	-2.86967
C	2.936707733	1.500255411	-1.77603
H	3.664624575	1.928805567	-1.06379
H	3.491722223	1.188406157	-2.6731
H	0.293632347	3.036510619	-1.20109
H	2.847840058	-0.37672672	-1.18622
O	4.106951265	-1.53797605	-1.32824
C	3.950032436	-2.83737363	-0.72348
H	3.046275435	-2.83052341	-0.09996
H	3.813564515	-3.56794366	-1.53266
C	5.398310141	-1.00415824	-0.99402
H	5.75790478	-0.42810573	-1.85405
H	5.302060592	-0.33291239	-0.12373
C	5.222499526	-3.08998598	0.069428
H	5.098693517	-2.74080402	1.103394
H	5.493920267	-4.14964203	0.090717
C	6.2407487	-2.2140709	-0.65362
H	6.594272034	-2.70192317	-1.5718
H	7.109371283	-1.96045199	-0.03848

SmI$_2$_EG_2_TSCF ENERGY_2

SCF ENERGY=-976.8179579

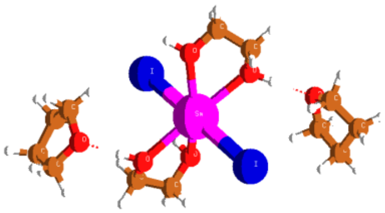

Sm	-0.69303	0.233481	0.196091
I	-3.44169	0.549314	2.357415
I	2.027208	0.532266	-2.04686
C	1.883616	2.005891	1.949268
H	2.300879	2.288549	2.927926
H	2.719241	1.951213	1.22818
C	0.901285	3.05946	1.513748
H	1.39281	4.041046	1.490226
H	0.06633	3.116371	2.223648
O	1.208372	0.760164	2.023218
H	1.873786	0.064	2.230458
O	0.32085	2.75014	0.247535
H	1.02979	2.69823	-0.41719
O	-2.56076	-0.1324	-1.68457
O	-0.92116	-2.24547	-0.9768
C	-1.81365	-2.34094	-2.09014
H	-2.14042	-3.37945	-2.23422
H	-1.31507	-1.99547	-3.00773
C	-3.00279	-1.4746	-1.80216
H	-3.49121	-1.8079	-0.87018
H	-3.727	-1.58005	-2.62285
H	-0.11009	-2.72398	-1.19331
H	-3.34839	0.459371	-1.70958
O	-4.80785	1.367786	-1.67866
O	3.196669	-1.01919	2.457046
C	-4.70696	2.720805	-1.1903
H	-3.69167	2.88467	-0.80189
H	-4.86463	3.397076	-2.04089
C	-5.88754	0.693823	-1.00933
H	-6.35208	0.010355	-1.72833
H	-5.48666	0.110207	-0.16386
C	-5.78108	2.871293	-0.12487
H	-5.36668	2.646074	0.866359

H	-6.19941	3.882013	-0.10136
C	-6.79501	1.801806	-0.51978
H	-7.44014	2.156439	-1.33436
H	-7.43003	1.482393	0.312358
C	4.251908	-0.45483	3.250759
H	4.645021	-1.23271	3.920518
H	3.809833	0.336446	3.867016
C	3.807399	-1.48443	1.249618
H	3.015877	-1.64171	0.50726
H	4.309567	-2.4462	1.44281
C	4.795332	-0.39469	0.892982
H	5.583458	-0.74172	0.217784
H	4.262744	0.425984	0.392682
C	5.322151	0.041095	2.267335
H	6.287429	-0.42663	2.488301
H	5.461152	1.125363	2.325501

SmI_2_EG_2_TSCF ENERGY_3

SCF ENERGY=-1209.1369214

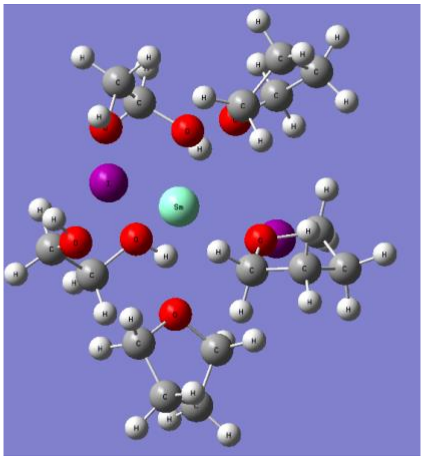

Sm	0.04511	-0.02703	0.47929
I	-3.55396	0.77275	0.12465
I	3.61585	-0.71869	-0.0245

O	-0.75431	-2.41096	-0.51398
C	0.09029	-3.57007	-0.63511
C	-1.962	-2.60443	-1.26697
C	-0.52784	-4.40942	-1.73621
H	1.11595	-3.24062	-0.84691
H	0.09634	-4.10518	0.32575
C	-2.00766	-4.08808	-1.57091
H	-2.80778	-2.24564	-0.66663
H	-1.9131	-1.99396	-2.18607
H	-0.29135	-5.47236	-1.63035
H	-0.17388	-4.07613	-2.72131
H	-2.42063	-4.6346	-0.71178
H	-2.61408	-4.32076	-2.45099
O	-0.08139	0.12112	-2.21267
C	0.68699	-0.75397	-3.0548
C	-0.85197	1.0253	-3.02192
C	0.63033	-0.13319	-4.4364
H	1.70112	-0.83668	-2.64021
H	0.22163	-1.75351	-3.04648
C	-0.76588	0.47823	-4.43352
H	-1.87127	1.07392	-2.61687
H	-0.40187	2.02985	-2.95925
H	0.79104	-0.86718	-5.23154
H	1.39119	0.65309	-4.53343
H	-1.52398	-0.30096	-4.59039
H	-0.91002	1.25459	-5.19079
O	0.77504	2.47329	-0.25227
C	1.88398	2.72406	-1.12894
C	-0.13559	3.5881	-0.28619
C	1.8594	4.21668	-1.38666
H	2.79797	2.36656	-0.63876
H	1.74816	2.14888	-2.06182
C	0.36174	4.49741	-1.39589
H	-1.15435	3.21054	-0.44901
H	-0.11151	4.08491	0.69394
H	2.36635	4.49064	-2.31666
H	2.3438	4.75108	-0.55873
H	-0.07292	4.20432	-2.36124
H	0.10858	5.54655	-1.21597
C	-0.20675	1.94442	3.43022
H	-0.45229	1.0498	4.02497
H	-0.49683	2.83461	4.00646
C	1.26967	1.98465	3.1662
H	1.8092	2.08626	4.1182
H	1.51288	2.8503	2.53175

C	0.16776	-2.38769	3.20229
C	-1.28226	-2.34997	2.81775
H	0.35473	-1.64903	3.99711
H	0.4177	-3.38579	3.58971
H	-1.8964	-2.64147	3.68138
H	-1.47263	-3.05895	1.99589
O	1.61753	0.77005	2.51245
H	2.51853	0.82384	2.15441
O	-0.8672	1.90724	2.17378
H	-1.81727	1.76225	2.29879
O	-1.57309	-1.01992	2.40832
H	-2.46617	-0.96906	2.02759
O	0.9315	-2.08114	2.04518
H	1.85077	-1.90886	2.29144

SmI_2_EG_3_TSCF ENERGY_3

SCF ENERGY=-1439.3053302

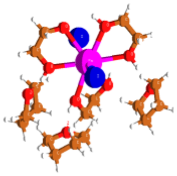

Sm	-0.406	-0.78904	-0.3362
I	-3.83481	-1.43218	0.627747
I	2.698179	-0.37243	-1.83422
C	-2.33496	1.128728	-2.71981
C	-2.13596	-0.18997	-3.41622
H	-3.32869	1.15105	-2.23694
H	-2.30932	1.937657	-3.46556
H	-1.15004	-0.21944	-3.89726
H	-2.89961	-0.32134	-4.1948
C	-0.54592	-4.33795	-0.5547
C	0.578482	-4.16008	0.423037
H	-1.50728	-4.35725	-0.0181
H	-0.42411	-5.28967	-1.0897
H	1.535969	-4.13946	-0.12354
H	0.596882	-5.01192	1.117977
C	0.01713	1.742876	2.217987
C	-0.97827	0.774856	2.78754
H	1.025597	1.507498	2.598938
H	-0.23975	2.758766	2.558907
H	-1.98974	1.017152	2.418995

H	-0.98225	0.856745	3.883262
O	-2.15418	-1.27586	-2.49188
O	-1.31255	1.286864	-1.75259
O	-0.0226	1.655595	0.804019
O	-0.60289	-0.53643	2.375332
O	0.36788	-2.93801	1.112528
O	-0.49254	-3.23351	-1.44918
C	-1.66614	4.608246	0.035012
O	-2.32377	3.49276	-0.57529
C	-3.26754	2.923345	0.352204
C	-3.18598	3.76676	1.616599
C	-2.63112	5.08983	1.097173
H	-0.71362	4.278587	0.485223
H	-1.44657	5.340359	-0.74966
H	-4.26239	2.959826	-0.11289
H	-3.00508	1.868389	0.522205
H	-4.15516	3.862975	2.114487
H	-2.48246	3.317573	2.331378
H	-3.42764	5.691982	0.64131
H	-2.14282	5.691159	1.870233
C	3.689329	-2.81477	1.550021
O	2.676474	-2.43242	2.488612
C	2.773503	-1.00851	2.563939
C	4.268362	-0.69532	2.530415
C	4.895494	-1.95798	1.906487
H	3.846059	-3.89345	1.646014
H	3.338813	-2.58852	0.526984
H	2.264333	-0.56963	1.685655
H	2.253039	-0.68416	3.469874
H	4.467812	0.205614	1.939948
H	4.655731	-0.52159	3.539544
H	5.503194	-1.73781	1.023188
H	5.532779	-2.47422	2.632158
C	3.240582	2.934707	0.61664
O	1.928936	3.49454	0.418218
C	2.019276	4.68636	-0.38319
C	3.496683	5.026838	-0.43502
C	4.134675	3.643837	-0.38015
H	3.552384	3.123899	1.656181
H	3.183658	1.849814	0.451055
H	1.614957	4.474388	-1.3852
H	1.405374	5.465814	0.084675
H	3.760048	5.599722	-1.32937
H	3.783627	5.614188	0.447384
H	4.068302	3.152606	-1.36064

H	5.183476	3.653198	-0.06896
H	0.639479	2.29308	0.456987
H	-1.48273	2.125781	-1.27035
H	1.107432	-2.79523	1.743766
H	-1.30349	-1.1547	2.63166
H	-1.26001	-3.21614	-2.03745
H	-3.00162	-1.26863	-2.00887

SmI_2_EDA_1_TSCF ENERGY_1

SCF ENERGY=-474.6244309

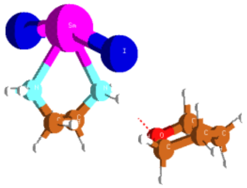

Sm	0.086204	0.226904	-1.9111
I	-3.35564	1.085175	-1.49362
I	3.542791	-0.32291	-1.25994
N	-0.36603	-1.16736	0.41616
H	-1.14731	-1.81739	0.354731
N	0.256874	1.732624	0.397072
C	-0.58644	-0.26119	1.554654
C	0.429425	0.861967	1.569135
H	-1.59576	0.164868	1.454894
H	-0.55365	-0.78897	2.519588
H	1.445979	0.441506	1.548532
H	0.323422	1.417675	2.513663
H	0.45272	-1.74299	0.612712
H	-0.62066	2.240648	0.539573
H	0.989073	2.440565	0.400071
C	-1.65277	4.623676	1.193645
O	-1.91103	3.338317	1.766792
C	-3.32034	3.188368	1.994565
C	-3.983	4.368592	1.3077
C	-2.90727	5.440822	1.432453
H	-0.75273	5.039292	1.663063
H	-1.45921	4.512725	0.111407
H	-3.64106	2.218323	1.592324
H	-3.50343	3.198248	3.080027

H	-4.16511	4.13428	0.249656
H	-4.93677	4.642231	1.769003
H	-3.01755	6.26329	0.719151
H	-2.90092	5.861451	2.447049

SmI_2_EDA_1_TSCF ENERGY_2

SCF ENERGY=-706.9320416

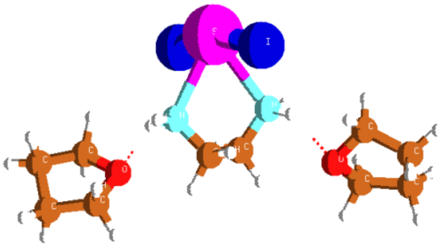

Sm	-0.25007	-0.15328	-1.84386
I	-3.64257	-0.50964	-0.99396
I	3.2873	0.051691	-1.92655
N	0.087156	-1.09839	0.733484
H	-0.46534	-1.93044	0.931582
N	-0.07149	1.789927	0.089413
C	-0.25391	-0.05016	1.706836
C	0.442573	1.2477	1.356926
H	-1.34264	0.104086	1.689339
H	0.01941	-0.33584	2.735186
H	1.521378	1.062028	1.251394
H	0.315698	1.956502	2.191099
H	1.06229	-1.35633	0.908698
H	-1.006	2.163278	0.286635
H	0.496563	2.589766	-0.18299
C	-1.78132	4.158443	2.097806
O	-2.39355	2.96367	1.595212
C	-3.67745	3.28044	1.040899
C	-3.67027	4.781072	0.831632
C	-2.84479	5.241095	2.027414
H	-1.4137	3.968804	3.113883
H	-0.91492	4.408666	1.462469
H	-3.81051	2.696319	0.121944
H	-4.46366	2.980328	1.751393
H	-3.15829	5.034194	-0.10685
H	-4.67716	5.20806	0.799609
H	-2.41792	6.242202	1.914093

H	-3.46083	5.231939	2.936213
C	3.541303	-2.37843	1.479824
O	2.694532	-1.58035	2.300129
C	2.158594	-2.47881	3.267024
C	3.270162	-3.48866	3.582557
C	4.294764	-3.267	2.45541
H	2.925121	-2.9835	0.786347
H	4.170561	-1.70874	0.88492
H	1.832402	-1.88886	4.129657
H	1.274884	-2.98926	2.842931
H	3.713865	-3.30332	4.565755
H	2.877665	-4.51082	3.58754
H	5.177529	-2.73828	2.832141
H	4.634505	-4.19886	1.992977

SmI_2_EDA_1_TSCF ENERGY_3

SCF ENERGY=-939.2435754

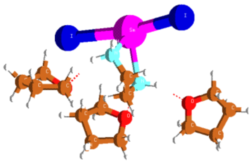

Sm	-0.11845	0.025369	-1.85745
I	-3.60186	-0.57673	-1.21852
I	3.385288	-0.48295	-2.20724
N	0.088136	-1.17184	0.611533
H	-0.45613	-2.03046	0.673757
N	0.098958	1.764932	0.263993
C	-0.37538	-0.23044	1.640429
C	0.3743	1.081083	1.53585
H	-1.45264	-0.0586	1.493424
H	-0.24599	-0.63041	2.659222
H	1.452936	0.874675	1.598812
H	0.113712	1.706581	2.406459
H	1.05071	-1.42426	0.850078
H	-0.81879	2.211075	0.360312
H	0.766981	2.532251	0.141095
C	3.33212	3.428341	-0.39817
O	2.168824	4.019586	0.182926
C	2.368809	4.123668	1.600216
C	3.627669	3.318675	1.934085
C	3.863228	2.496392	0.668591

H	3.028203	2.925847	-1.32521
H	4.068506	4.209568	-0.65022
H	2.463704	5.181726	1.879656
H	1.468125	3.726727	2.091701
H	4.475803	3.991266	2.110837
H	3.499301	2.700912	2.828881
H	4.911128	2.220453	0.513702
H	3.270581	1.569839	0.680937
C	-1.69335	4.277947	1.92627
O	-2.30165	3.077585	1.437835
C	-3.64673	3.351369	1.020397
C	-3.76345	4.863326	0.974167
C	-2.82617	5.272118	2.104655
H	-1.1569	4.04754	2.855713
H	-0.96162	4.642691	1.1841
H	-3.81799	2.858822	0.055415
H	-4.34279	2.917031	1.754392
H	-3.39169	5.24787	0.014993
H	-4.79266	5.209998	1.106872
H	-2.48729	6.310868	2.046435
H	-3.31556	5.122304	3.076092
C	3.580223	-2.24114	1.573645
O	2.61877	-1.52924	2.346414
C	2.079809	-2.48711	3.254235
C	3.223916	-3.44948	3.604137
C	4.320956	-3.09949	2.584414
H	3.06521	-2.86798	0.820501
H	4.202519	-1.51101	1.045754
H	1.674809	-1.94266	4.113556
H	1.249774	-3.02876	2.766338
H	3.572668	-3.30766	4.631758
H	2.897018	-4.49032	3.510018
H	5.116466	-2.51149	3.056233
H	4.78148	-3.98068	2.127274

SmI_2_EDA_2_TSCF ENERGY_1

SCF ENERGY= -665.0496255

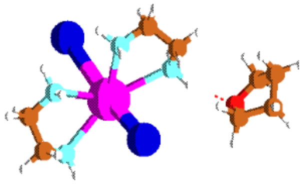

Sm	-0.15902	-0.34394	-0.93255
I	2.374589	-2.28176	0.649533
I	-2.75901	2.057116	-1.27527
C	-2.59881	-2.22856	0.952169
H	-3.06879	-2.64491	1.856719
H	-3.41523	-1.86341	0.311169
C	-1.85481	-3.33326	0.2311
H	-2.49505	-4.22824	0.189994
H	-0.95987	-3.60492	0.809468
C	1.290419	2.475791	0.803392
H	1.7147	3.013035	1.666005
H	0.644761	3.189023	0.269931
C	2.416262	2.044869	-0.11439
H	2.98979	1.237352	0.368156
H	3.109805	2.892378	-0.247
N	-1.70653	-1.1019	1.24374
H	-2.2478	-0.31829	1.603453
H	-1.06846	-1.36954	1.992864
N	-1.41693	-2.89108	-1.09842
H	-0.82821	-3.61046	-1.51289
H	-2.23392	-2.82847	-1.70514
N	1.90059	1.532736	-1.38918
H	1.528584	2.314703	-1.92767
H	2.68853	1.163725	-1.92657
N	0.468518	1.327773	1.198925
H	0.986049	0.759601	1.869613
H	-0.3634	1.653649	1.686298
C	5.309211	-0.02937	-1.45243
O	4.631011	0.779996	-2.42172
C	5.28551	2.052072	-2.52459
C	6.354387	2.070474	-1.44335
C	6.684219	0.588333	-1.30666
H	4.757626	0.000959	-0.49678
H	5.312948	-1.06474	-1.8111
H	5.724734	2.139293	-3.52939
H	4.536641	2.849106	-2.41219
H	7.213228	2.691155	-1.71599
H	5.943988	2.453252	-0.49905
H	7.337647	0.262643	-2.12707

H	7.165494	0.330372	-0.35854

SmI_2_EDA_2_TSCF ENERGY_2

SCF ENERGY=-897.3600447

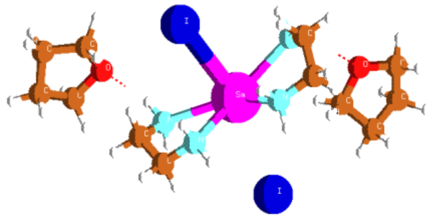

Sm	-0.35788	-0.2086	-0.70423
I	2.28683	-2.30772	0.54299
I	-2.57762	2.642746	-0.79372
C	-2.69099	-2.1337	1.251657
H	-3.13798	-2.54607	2.169765
H	-3.52414	-1.78933	0.620027
C	-1.94127	-3.23628	0.532379
H	-2.58113	-4.13191	0.495053
H	-1.04984	-3.50629	1.117859
C	1.356574	2.490695	0.94226
H	1.861345	3.013417	1.769885
H	0.688938	3.220969	0.461448
C	2.397994	2.030284	-0.05676
H	2.990483	1.215863	0.389211
H	3.095602	2.862603	-0.24887
N	-1.81129	-0.98864	1.51399
H	-2.34999	-0.22519	1.918089
H	-1.12509	-1.25103	2.221968
N	-1.50498	-2.80233	-0.80141
H	-0.84421	-3.48305	-1.17016
H	-2.31392	-2.82817	-1.42676
N	1.77997	1.517655	-1.28566
H	1.414999	2.306087	-1.81984
H	2.517486	1.101154	-1.8607
N	0.536985	1.3637	1.401591
H	1.105182	0.76456	2.000512
H	-0.21447	1.71366	1.992515
C	5.153778	-0.11517	-1.59444
O	4.399718	0.681173	-2.51791
C	5.049458	1.947899	-2.69616

C	6.20168	1.979324	-1.70498
C	6.538235	0.498048	-1.57498
H	4.684649	-0.06433	-0.59663
H	5.120293	-1.1572	-1.93155
H	5.407297	2.014695	-3.7343
H	4.31475	2.750418	-2.53819
H	7.036758	2.594304	-2.05392
H	5.868012	2.375237	-0.73621
H	7.118972	0.159322	-2.44344
H	7.097212	0.251011	-0.66741
C	-5.28946	-2.57146	-3.05575
O	-4.08338	-2.2567	-2.3464
C	-3.89911	-0.83766	-2.32066
C	-5.19466	-0.23138	-2.82744
C	-5.66861	-1.30537	-3.79966
H	-5.09413	-3.42988	-3.7084
H	-6.0689	-2.85698	-2.33227
H	-3.64937	-0.52704	-1.29539
H	-3.05508	-0.57258	-2.98158
H	-5.9103	-0.11619	-2.00265
H	-5.04265	0.750449	-3.28575
H	-6.73775	-1.25395	-4.02651
H	-5.11241	-1.23613	-4.74407

SmI_2_EDA_2_TSCF ENERGY_3

SCF ENERGY=-1129.6727531

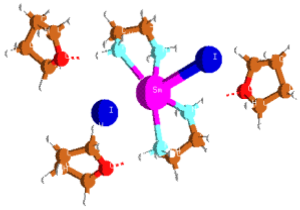

Sm	-0.49086	-0.54315	-0.96578
I	1.93044	-2.44814	0.915121
I	-2.89678	1.96444	-1.66296
C	-2.97761	-2.44744	0.746004
H	-3.53566	-2.86516	1.600168
H	-3.7276	-2.11047	0.01276
C	-2.13363	-3.54007	0.119581
H	-2.73575	-4.45921	0.042182
H	-1.28887	-3.76748	0.786562
C	0.97898	2.315532	0.658304
H	1.360343	2.906077	1.506209
H	0.418539	3.009874	0.014389
C	2.153872	1.753247	-0.11779
H	2.646311	0.9758	0.487209
H	2.894597	2.555398	-0.27162
N	-2.15925	-1.28835	1.118039
H	-2.77976	-0.54537	1.450138
H	-1.57149	-1.54362	1.911477
N	-1.58989	-3.11098	-1.17357
H	-0.8798	-3.77231	-1.50017
H	-2.33693	-3.14058	-1.8661
N	1.717057	1.138806	-1.37641
H	1.447229	1.877903	-2.02496
H	2.508011	0.655103	-1.81101
N	0.067312	1.244948	1.075881
H	0.504877	0.706942	1.823739
H	-0.77638	1.648611	1.478476
C	-5.58649	0.035977	1.064062
O	-4.52956	0.411983	1.952013
C	-4.63947	1.808171	2.264345
C	-5.78599	2.346812	1.423943
C	-6.64797	1.104972	1.226871
H	-5.20606	0.013489	0.027546
H	-5.922	-0.97172	1.337254

H	-4.84122	1.906649	3.3414
H	-3.68187	2.300776	2.04644
H	-6.3098	3.173463	1.913689
H	-5.41012	2.700563	0.45497
H	-7.25244	0.907334	2.122404
H	-7.31829	1.169547	0.364242
C	4.963688	-0.6748	-1.48805
O	4.268826	0.02355	-2.53209
C	4.949446	1.254425	-2.81756
C	6.026362	1.399756	-1.75587
C	6.345812	-0.05692	-1.44154
H	4.429869	-0.5307	-0.53237
H	4.952475	-1.74611	-1.72186
H	5.38291	1.191498	-3.82668
H	4.216832	2.074179	-2.81342
H	6.88665	1.976183	-2.1091
H	5.625211	1.897685	-0.8626
H	6.983504	-0.4861	-2.22584
H	6.840223	-0.20118	-0.4763
C	1.460584	-5.65545	-1.78963
O	0.639187	-4.70253	-2.48688
C	1.476169	-3.79979	-3.20832
C	2.76808	-4.55576	-3.43618
C	2.904025	-5.28954	-2.10663
H	1.233244	-5.60777	-0.71569
H	1.19967	-6.65857	-2.15571
H	0.952712	-3.50769	-4.12606
H	1.667988	-2.89359	-2.60169
H	2.648797	-5.26656	-4.26497
H	3.611925	-3.89692	-3.66319
H	3.556335	-6.16689	-2.15159
H	3.297098	-4.60489	-1.34251

SmI_2_EDA_3_TSCF ENERGY_1

SCF ENERGY=-855.4650511

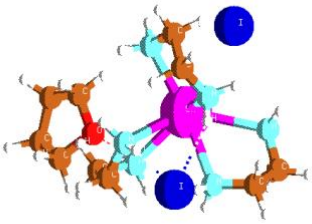

Sm	-0.73493	0.742932	0.356562
I	4.230406	-0.97032	-0.59727
I	-4.15169	-0.51763	0.552798
N	0.379018	-1.61769	-0.61225
H	1.376894	-1.66332	-0.39684
N	-1.48329	0.00551	-2.27431
C	0.198119	-1.77303	-2.06022
C	-1.21727	-1.42524	-2.47316
H	0.89915	-1.09262	-2.56686
H	0.439761	-2.78842	-2.41048
H	-1.92862	-1.99094	-1.85265
H	-1.37822	-1.7448	-3.51471
H	-0.04188	-2.42022	-0.145
H	-0.99407	0.529189	-2.99853
H	-2.47384	0.182172	-2.43082
N	1.38089	1.606735	-1.22281
H	2.073898	0.854919	-1.24009
N	0.081652	3.465611	0.696264
C	2.005159	2.832103	-0.71601
C	0.956896	3.882574	-0.40536
H	2.554113	2.583575	0.204364
H	2.744978	3.254389	-1.41424
H	0.328898	4.033426	-1.29733
H	1.458693	4.844215	-0.20866
H	1.112061	1.753735	-2.19445
H	0.590656	3.558431	1.573968
H	-0.70233	4.119621	0.747777
N	-0.93614	-0.81396	2.737815
H	-1.78341	-0.64371	3.275404
N	1.604692	0.366786	1.883398
C	0.239259	-0.7219	3.608444
C	1.514573	-0.77928	2.793988
H	0.196044	0.239881	4.140144
H	0.266697	-1.50873	4.378727
H	1.518286	-1.69862	2.189315
H	2.377528	-0.85252	3.474632
H	-1.0291	-1.77202	2.400929
H	1.839696	1.194149	2.431027
H	2.399847	0.223444	1.256244
C	-3.03085	5.613586	-0.71331
O	-1.97195	5.623097	0.240504
C	-1.27639	6.842003	0.001155
C	-2.37402	7.879323	-0.18601
C	-3.52535	7.060889	-0.80232

H	-2.63873	5.271868	-1.68702
H	-3.78687	4.899298	-0.37308
H	-0.60908	7.026124	0.849693
H	-0.66482	6.747507	-0.91441
H	-2.66968	8.293748	0.783948
H	-2.05159	8.710507	-0.82062
H	-4.45256	7.199058	-0.23755
H	-3.72753	7.34296	-1.84052

SmI_2_EDA_3_TSCF ENERGY_2

SCF ENERGY=-1087.7787154

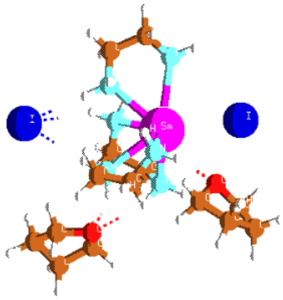

Sm	-0.87805	0.574961	0.28795
I	4.064213	-0.86026	-1.24635
I	-4.18898	-0.86935	0.817086
N	0.266741	-1.77308	-0.70363
H	1.279311	-1.78156	-0.56849
N	-1.79591	-0.31208	-2.28032
C	-0.02743	-2.01366	-2.12136
C	-1.48602	-1.73878	-2.42808
H	0.604206	-1.34112	-2.72491
H	0.218814	-3.04032	-2.43574
H	-2.118	-2.29976	-1.72311
H	-1.71776	-2.11929	-3.4368
H	-0.0916	-2.55909	-0.16167
H	-1.32633	0.200208	-3.02812
H	-2.79478	-0.17248	-2.41783
N	0.973073	1.419605	-1.5585
H	1.703733	0.706937	-1.62142
N	-0.0921	3.313412	0.416687
C	1.58882	2.716968	-1.27774
C	0.543297	3.724757	-0.84026
H	2.317362	2.582548	-0.46298
H	2.15	3.118183	-2.13834
H	-0.24085	3.790441	-1.61084

H	1.007101	4.723239	-0.77715
H	0.538442	1.436249	-2.48152
H	0.576006	3.436595	1.176111
H	-0.86794	3.949074	0.61616
N	-0.74539	-0.84274	2.756674
H	-1.52511	-0.66774	3.386843
N	1.620149	0.371362	1.545682
C	0.524392	-0.68053	3.471604
C	1.689781	-0.73415	2.505175
H	0.515839	0.299018	3.971262
H	0.673216	-1.43663	4.258523
H	1.648187	-1.6804	1.944341
H	2.633213	-0.75008	3.073413
H	-0.84772	-1.81987	2.482374
H	1.869724	1.235047	2.026022
H	2.339951	0.239801	0.83195
C	-0.01172	-0.36852	-5.46575
O	0.494366	0.580123	-4.51768
C	1.900519	0.781263	-4.75021
C	2.315588	-0.29659	-5.73192
C	1.042149	-0.46888	-6.55097
H	-0.14412	-1.34465	-4.96903
H	-0.99086	-0.02189	-5.81769
H	2.047567	1.788581	-5.16978
H	2.434872	0.723242	-3.79262
H	3.190721	-0.01053	-6.32264
H	2.550385	-1.22732	-5.19635
H	0.934438	0.353735	-7.27052
H	0.991822	-1.41537	-7.09748
C	-3.39763	5.488609	-0.23978
O	-2.20048	5.469285	0.534661
C	-1.47072	6.61628	0.110672
C	-2.5119	7.722435	0.031331
C	-3.79285	6.964225	-0.36521
H	-3.1941	5.050913	-1.2324
H	-4.13744	4.863059	0.268846
H	-0.66565	6.794296	0.831151
H	-1.02231	6.424644	-0.88106
H	-2.63081	8.1949	1.012557
H	-2.23413	8.502215	-0.68423
H	-4.62164	7.213094	0.304652
H	-4.1139	7.197232	-1.38536

SmI_2_EDA_3_TSCF ENERGY_3

SCF ENERGY=-1320.090225

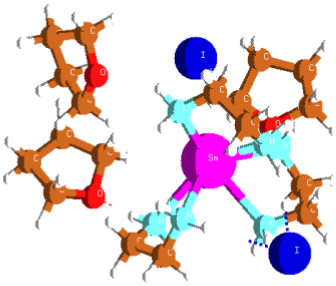

Sm	0.569728	0.079061	0.020758
I	5.854187	0.56293	-0.09845
I	-2.39349	-1.02451	-1.76006
N	2.515073	-0.31637	-1.91487
H	3.444553	-0.40426	-1.50024
N	0.420623	1.75803	-2.22426
C	2.542296	0.743135	-2.92959
C	1.13948	1.139423	-3.34309
H	3.050711	1.616761	-2.49331
H	3.119143	0.460267	-3.82403
H	0.584059	0.241088	-3.65294
H	1.197175	1.795502	-4.22628
H	2.327109	-1.20718	-2.3738
H	0.818471	2.682534	-2.06016
H	-0.5516	1.940406	-2.48146
N	2.275307	2.17801	0.666904
H	3.229213	1.934428	0.393085
N	0.164	1.271582	2.554122
C	2.245296	2.504878	2.09569
C	0.820147	2.582765	2.608173
H	2.780877	1.710284	2.636753
H	2.764101	3.447752	2.329083
H	0.249948	3.279754	1.97314
H	0.826285	3.013954	3.622475
H	2.04343	3.014352	0.132565
H	0.632699	0.6506	3.214052
H	-0.7987	1.344481	2.894174
N	0.649814	-2.71758	0.071514
H	-0.2758	-3.08963	0.296312
N	2.714515	-1.08509	1.389208
C	1.624581	-3.25662	1.023201
C	2.925057	-2.47866	0.978341
H	1.193538	-3.17256	2.032369
H	1.833507	-4.32696	0.861294

H	3.311628	-2.47769	-0.05208
H	3.679141	-2.99458	1.593521
H	0.866762	-3.0489	-0.86797
H	2.592547	-1.06083	2.401228
H	3.569537	-0.55438	1.205222
C	-3.53454	0.704723	2.543883
O	-2.72022	1.439048	3.454026
C	-3.07382	2.79934	3.22645
C	-4.59397	2.800378	3.075847
C	-4.91126	1.357091	2.630807
H	-3.11773	0.799264	1.522081
H	-3.50862	-0.34974	2.83462
H	-2.69499	3.395182	4.062562
H	-2.58722	3.15157	2.296696
H	-5.07758	3.029128	4.031259
H	-4.92539	3.552273	2.350997
H	-5.52913	0.848064	3.377703
H	-5.44162	1.309755	1.674213
O	-1.64024	-4.33534	1.191911
O	-2.18709	3.250894	-2.14161
C	-3.04543	-4.05456	1.114731
C	-1.34047	-5.49975	0.410376
C	-2.55601	3.128519	-0.76577
C	-3.3445	3.069876	-2.96565
C	-2.6774	-6.13775	0.084345
C	-3.57636	-4.91144	-0.01864
C	-4.52477	3.363524	-2.06406
C	-4.03785	2.776116	-0.74358
H	-3.51615	-4.32024	2.074226
H	-3.17666	-2.97765	0.946941
H	-0.81692	-5.18881	-0.51005
H	-0.6678	-6.14429	0.989744
H	-2.64406	-6.73887	-0.82928
H	-3.00539	-6.7833	0.910002
H	-3.4218	-4.40213	-0.98045
H	-4.64247	-5.1344	0.086637
H	-3.25668	3.743849	-3.82503
H	-3.37526	2.030993	-3.33422
H	-5.45506	2.912794	-2.42378
H	-4.67557	4.447463	-1.97358
H	-4.55143	3.18661	0.133113
H	-4.16933	1.684861	-0.74851
H	-1.92909	2.353577	-0.29156
H	-2.35248	4.086861	-0.26287
